# Construction and Evaluation of Chitosan-Based Nanoparticles for Oral Administration of Exenatide in Type 2 Diabetic Rats

**DOI:** 10.3390/polym14112181

**Published:** 2022-05-27

**Authors:** Jian-Miao Yang, Lin-Jie Wu, Meng-Ting Lin, Yi-Ying Lu, Tian-Tian Wang, Min Han, Bin Zhang, Dong-Hang Xu

**Affiliations:** 1 Department of Pharmacy, The Second Affiliated Hospital, College of Medicine, Zhejiang University, Hangzhou 310009, China; yangjm@enzemed.com (J.-M.Y.); 15574881635@163.com (T.-T.W.); 2 Taizhou Hospital of Zhejiang Province, Zhejiang University, Taizhou 317099, China; 3 Institute of Pharmaceutics, College of Pharmaceutical Sciences, Zhejiang University, Hangzhou 310058, China; 22119114@zju.edu.cn (L.-J.W.); 15700060560@163.com (M.-T.L.); 21919089@zju.edu.cn (Y.-Y.L.); 4 Zhejiang Strong Pharmaceutical Co., Ltd., Hangzhou 311500, China

**Keywords:** exenatide, chitosan-based nanocarrier, sodium alginate, hypoglycemia, trimethyl chitosan

## Abstract

Oral delivery of therapeutic peptides has been a daunting challenge due to poor transport across the tight junctions and susceptibility to enzymatic degradation in the gastrointestinal tract. Numerous advancement in nanomedicine has been made for the effective delivery of protein and peptide. Owing to the superior performance of chitosan in opening intercellular tight junctions of epithelium and excellent mucoadhesive properties, chitosan-based nanocarriers have recently garnered considerable attention, which was formulated in this paper to orally deliver the GLP-1 drug (Exenatide). Against this backdrop, we used chitosan (CS) polymers to encapsulate the exenatide, sodium tripolyphosphate (TPP) as the cross-linking agent and coated the exterior with sodium alginate (ALG) to impart the stability in an acidic environment. The chitosan/alginate nanoparticles (CS-TPP-ALG) functioned as a protective exenatide carrier, realized efficient cellular uptake and controlled release, leading to a steady hypoglycemic effect and a good oral bioavailability in vivo. Trimethyl chitosan (TMC), a chitosan derivative with stronger positive electrical properties was additionally selected as a substitute for chitosan to construct the TMC-TPP-ALG nanoparticle, and its oral peptide delivery capacity was explored in terms of both characterization and pharmacodynamics studies. Overall, our study demonstrated that functional chitosan/alginate nanoparticles can protect proteins from enzymatic degradation and enhance oral absorption, which presents important research value and application prospects.

## 1. Introduction

Global peptide markets are flourishing rapidly due to the attractive properties of peptides such as specificity, efficacy, potency, and low toxicity [1,2]. In therapeutic applications, most peptide/protein drugs are injected subcutaneously, resulting in high costs and reduced compliance [3,4,5]. Hence, a creative drug delivery method with wide acceptance and great convenience is much desired, among which the most preferred oral delivery system received the widest attention [6]. Despite that, this alternative route of administration of peptides is beset by some unfavorable constraints. The harsh physiological environment of the gastrointestinal tract, for example, incurs enzymatic degradation and inactivation susceptible to protein/peptide drugs [7,8]. The hydrophilic property and macromolecular structure of the protein, as well as the viscous mucous layer, also engender formidable barriers to the absorption and penetration across the intestinal membrane [9,10]. Currently, addressing these issues to enhance the absorption of orally administrated peptide-based drugs remains a significant challenge.

**Figure 1:**
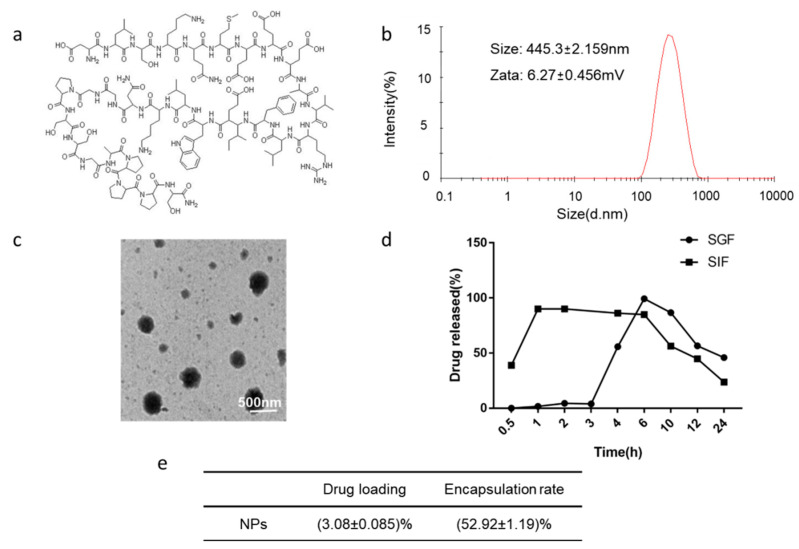
(**a**) The chemical structure of exenatide. (**b**) The size distribution and zeta potential of CS-TPP-ALG. (**c**) TEM image of CS-TPP-ALG. (**d**) In vitro drug release profile of exenatide nano-preparation. (**e**) Encapsulation rate and drug loading capacity of exenatide nanoparticles.

Exenatide (Figure 1a) is an analog of glucagon-like peptide-1 (GLP-1) that has been used in the treatment of type 2 diabetes mellitus (T2DM) [11]. As a GLP-1 receptor agonist, exenatide exhibits great efficacy in stimulating insulin secretion and attenuating the blood glucose level. Furthermore, exenatide can also effectively induces satiety, reduce body weight, and may have additional cardiovascular protective effects as compared with the traditional hypoglycemic drug insulin [12]. ORMD-0901 (exenatide-4) is an oral exenatide-based capsule developed by Oramed Pharmaceuticals, which is currently in Phase 2 clinical trials [13], while not yet available in markets, which reflects that the development of oral delivery systems for exenatide remains limited. Though various improvements have been reported, the natural polymeric nanoparticles, with better capacity and physical stability, appear to present more predominant applications in the oral administration of bioactive substances [14,15]. Chitosan (CS) is a biodegradable, biocompatible, and adhesive cationic polymer that readily forms complexes or nanoparticles in aqueous media, possessing the ability to encapsulate the drug molecules [15]. Attributed to its electrostatic interaction with the negatively charged mucin, chitosan is capable of transiently opening the tight junctions between epithelial cells, promoting the paracellular absorption of drugs [16,17]. Considering the functional properties of low toxicity, high storage and special cellular affinity, nanoparticles formulated with chitosan for oral administration of peptides hold great promise [18,19,20,21]. Nevertheless, chitosan has limited utility due to its deprotonation and dissolution under gastric pH conditions, which has prompted the exploitation of acid-resistant polymer for sustained, controlled, and oral delivery [22,23]. Given the tailored properties of controlled release of sodium alginate (ALG), it can protect drugs from degradation in the acidic environment and, more importantly, can enhance the oral bioavailability of drugs, thereby providing an ideal candidate for the enteric coating of nanoparticles [23,24]. Ascribe to the strong electrostatic interaction between alginate and chitosan, the nanoparticles are capable of shrinking and gelling at low pH, aiming to protect the peptide from the aggressive stomach environment [25]. In general, an oral administration carrier based on alginate-coated chitosan nanoparticles, with enhanced permeability, excellent stability, and high drug payload, can be engineered to orally deliver a host of therapeutic peptides and proteins such as exenatide, presenting extensive application prospects [26].

In the present study, chitosan nanoparticles were prepared to encapsulate hydrophilic exenatide, using sodium tripolyphosphate (TPP) as the cross-linking agent, and sodium alginate was further coated to modify the stability and bioavailability of exenatide loaded nanoparticles. Based on the conceived advantage of the positive charge of chitosan in promoting the absorption of peptide drugs, derivative trimethyl chitosan (TMC) with stronger positive electrical properties was further constructed as a vehicle for the delivery of exenatide to explore the impact of structural, electrical and other properties of different materials on oral administration of peptide drugs, providing a practicable format for the preparation of exenatide as an oral formulation for the treatment of type 2 diabetes mellitus.

## 2. Materials and Methods

### 2.1. Materials

Exenatide was purchased from Shanghai GL Biochem Co., Ltd. (Shanghai, China). Chitosan (Mw: 50 k) was purchased from Zhejiang Golden-Shell Pharmaceutical Co., Ltd. (Zhejiang, China). Sodium alginate was purchased from Shanghai Aladdin Reagent Co., Ltd. (Shanghai, China). Sodium citrate and Citric acid were purchased from Sinopharma (Shanghai, China). Insulin was purchased from Jiangsu Wanbang Pharmaceutical Co., Ltd. (Jiangsu, China). Streptozotocin was purchased from Shanghai McLean Biochemical Technology Co., Ltd. (Shanghai, China). Methyl alcohol (HPLC) was purchased from Tianjin Siyou Chemicals Co., Ltd. (Tianjin, China). High-fat diets were purchased from Shanghai SLAC Laboratory Animal Co., Ltd. (Shanghai, China). DAPI staining solution was purchased from Jiangsu Keygen Biotech Co., Ltd. (Jiangsu, China). Other solvents and reagents are analytical or chromatographic pure.

### 2.2. Cell Culture

Human colon cancer Caco-2, HT-29, and Human Burkitt’s lymphoma cells Raji-B were purchased from the Cell Bank of the Chinese Academy of Sciences (Shanghai, China). Caco-2 was cultured in high-sugar Dulbecco’s modified Eagle’s medium (DMEM) supplemented with 10% Fetal Bovine Serum (FBS), 1% penicillin-streptomycin mixed solution, and 1% nonessential amino acid. HT-29 and Raji B cells were cultured in 1640 medium with 10% FBS and 1% penicillin-streptomycin mixed solution. Cells were digested with trypsin containing 0.02% EDTA. Passage could be performed when the cells grew to 80~90%.

### 2.3. Experimental Animals

Sprague-Dawley (SD) rats (male, 200 g) were purchased from Shanghai Laboratory Animal Center (SLAC) (Shanghai, China). The animals were maintained under standard housing conditions and all related experiments were conducted according to guidelines that have been evaluated and approved by the ethics committee of Zhejiang University (ethical approval code: ZJU20210258).

### 2.4. Quantification of Exenatide

The quantification of exenatide was performed via HPLC (1200, Agilent, CA, USA). Specifically, samples containing exenatide were analyzed on a Diamonsil C18 column (5 µm, 250 × 4.6 mm) at 25 °C with gradient elution (1.0 mL/min): 0~30 min; linear-gradient: from 100% A/0% B to 0% A/100% B (mobile phase A: 0.1% TFA aqueous solution; mobile phase B: 0.1% TFA acetonitrile solution) with signal determination at 220 nm (Figure S1a).

An appropriate amount of exenatide was dissolved in methanol and analyzed by HPLC after dilution to evaluate its specificity (Figure S1c,d).

A series of exenatide solutions with different concentrations were prepared and filtered by a 0.22 µm microporous membrane. Recorded the peak time and peak area of exenatide. The calibration curve was established with exenatide in a range from 12.5 to 1600 μg/mL (Figure S1b).

### 2.5. Preparation and Characterization of Exenatide Nano-Preparation

Chitosan-based nanoparticles were prepared by ionotropic gelation method using TPP as a polyanionic crosslinking agent [27]. Exenatide (10 mg) was dissolved in 100 μL of water, into which 20 mL of chitosan (2 mg/mL) was added at pH6.0. After stirring overnight, 2 mL of TPP solution (0.7 mg/mL) was added for reaction of 10 min to prepare CS-TPP nano-preparations. After completion of the reaction, 100 mL of sodium alginate (0.3 mg/mL) was added and incubated for an additional 30 min to obtain the CS-TPP-ALG nano-preparations.

Trimethyl chitosan-tripolyphosphate (TMC-TPP) nano-preparation was prepared according to the above steps of CS-TPP synthesis, where CS was replaced with derivative Trimethyl chitosan (TMC), which was obtained by methylation of chitosan with sodium iodide and methane iodide in DMSO. Subsequently, sodium alginate (0.3 mg/mL) was added and incubated for an additional 60 min to obtain the trimethyl chitosan-tripolyphosphate-sodium alginate (TMC-TPP-ALG) nano-preparations.

To construct Rhodamine-labeled nanoformulations for subsequent experiments, 80 mg of Rhodamine B was first activated in a mixture of 20 mL N,N-Dimethylformamide (DMF), 400 mg 2-(1H-Benzotriazole-1-yl)-1,1,3,3-tetramethyluronium tetrafluoroborate (TBTU) and 80 μL N-Ethyldiisopropylamine (DIEA) for 2 h, and then 20 mg/mL of chitosan solution was added, followed by an overnight reaction to obtain Rhodamine-labeled chitosan. The synthetic procedures of Rho-CS-TPP-ALG NPs (Rho-NPs) also refer to the above steps of CS-TPP-ALG synthesis.

Size distribution, zeta potential, and PDI values of CS-TPP, CS-TPP-ALG, TMC-TPP-ALG and Rho-NPs were evaluated using a Malvern particle size analyzer (Zetasizer90, Malvern, Shanghai, China). The freshly prepared nano-preparation was diluted to an appropriate concentration, and then dropped onto a 300-mech copper net. After natural drying, the morphology of the exenatide nano-preparation was observed under the transmission electron microscope (TEM, JEM-1400 flash, Jeol, Japan).

### 2.6. In Vitro Release Assay

Release studies were performed via the dialysis membrane method. Firstly, CS-TPP-ALG NPs were added into a dialysis membrane bag, which was immersed in the release medium of 4 mL simulated gastric fluid (pH1.2) or simulated intestinal fluid (pH 7.4) and subsequently placed in a 37 °C constant temperature oscillating chamber. A total of 0.4 mL of dialysis external fluid was extracted at 0.5 h, 1 h, 2 h, 3 h, 4 h, 6 h, 10 h, 12 h, and 24 h, respectively, and supplemented with equal amounts of the same fresh medium. The samples were centrifuged at 13,000 rpm for 20 min to collect the supernatant, which was taken for HPLC injection. The cumulative release was calculated by substituting the regression curve equation, and the drug release curves were plotted.

### 2.7. Determination of Encapsulation Rate and Loading Capacity

1 mL CS-TPP-ALG solution was taken and centrifuged at 15,000 rpm/min at 4 °C for 60 min. The supernatant was extracted for HPLC examination to determine the drug concentration. The encapsulation rate (EE%) and drug loading capacity (DL%) were calculated according to the following formula.

EE% = (Total amount of Ex) − (Free Ex)/(Total amount of Ex) × 100%(1)

DL% = (Total amount of Ex) − (Free Ex)/(Weight of nanoparticles) × 100%(2)

Ex represents the exenatide.

### 2.8. Cytotoxicity Study

The cytotoxicity of chitosan-based nanoparticles was measured by MTT assay [28]. Caco-2, HT-29, and Raji-B cells at the logarithmic growth stage were inoculated on 96-well plates at 5 × 10^3^ cells per well, respectively. The cells were cultured at 37 °C, 5% CO_2_ for 48 h, then the culture medium was removed and 150 μL of drug-containing culture medium was added to the final concentrations of 1, 0.5, 0.25, 0.125, 0.06, and 0.03 μM. After incubation for 4 h, 20 μL of MTT solution (5 mg/mL) was added to each well, which was incubated for an additional 4 h. Then, the culture medium was discarded and replaced with 150 μL dimethyl sulfoxide. The plates were placed on the shaker and oscillated for 10 min.

Caco-2 cells at the logarithmic growth stage were inoculated on 96-well plates at 2 × 10^3^ cells per well. The cells were cultured at 37 °C, 5% CO2 for 48 h, then the culture medium was removed and 150 μL of drug-containing culture medium was added to the final concentrations of 25, 12.5, 6.25, 3.12, and 1.56 μg/mL. After incubation for 24 h or 72 h, 20 μL of MTT solution (5 mg/mL) was added to each well, which was incubated for an additional 4 h. Then, the culture medium was discarded and replaced with 150 μL dimethyl sulfoxide. The plates were placed on the shaker and oscillated for 10 min.

The absorbance was measured at 570 nm using an enzyme marker (ST360, KHB, Shanghai, China). Cell viability was calculated as a percentage of the absorbance relative to that of the control.

### 2.9. In Vitro Cellular Uptake of Exenatide Nano-Preparation

Caco-2 cells at the logarithmic growth stage were inoculated in cell plates at a density of 2 × 10^5^ cells/mL. The Caco-2/HT-29 cell model was established by inoculating Caco-2 and HT-29 cells at a population ratio of 7:3. The medium was changed every other day for the first week and daily from the second week. A total of 10^5^ cells/well of Raji-B cells were added to establish Caco-2/HT-29/Raji-B three-cell model. After 14 days of incubation at 37 °C with 5% CO2, the medium was removed and the culture medium containing Rhodamine B, Rhodamine-CS-TPP (Rho-CS), or Rho-CS-TPP-ALG NPs (Rho-NPs) was added with a final drug fluorescence concentration of 1 μg/mL. After incubation for 4 h, the drug-containing culture medium was discarded and the cells were washed with PBS three times. Subsequently, trypsin containing 0.2% EDTA was added to digest the cells, which were collected, centrifuged, and re-suspended with 0.5 mL of PBS. The intracellular drug uptake was determined by flow cytometry (FC500MCL, Beckman, MA, USA).

### 2.10. Intracellular Distribution of Exenatide Nano-Preparation

Caco-2 cells at the logarithmic growth stage were inoculated in 12-well plates at a density of 2 × 10^5^ cells/mL. The Caco-2/HT-29 cell model was established by inoculating Caco-2 and HT-29 cells at a population ratio of 7:3. The medium was changed every other day for the first week and daily from the second week. After 14 days of incubation at 37 °C with 5% CO2, the medium was removed and the culture medium containing Rhodamine B, Rho-CS, or Rho-CS-TPP-ALG NPs was added with a final drug fluorescence concentration of 2 μg/mL. After incubation for 4 h, the drug-containing culture medium was discarded. The cells were washed three times with PBS and fixed with paraformaldehyde solution for 10 min and the nuclei were subsequently stained with DAPI. Then, the cells were incubated for 10 min at room temperature, washed three times with PBS, and observed under a fluorescence microscope (LSM 800, Zeiss, Oberkochen, Germany).

### 2.11. Effect of Exenatide Nano-Preparation on Fluidity of Caco-2 Cell Membrane

Caco-2 cells at the logarithmic growth stage were inoculated in confocal dishes at a density of 2 × 10^5^ cells/mL. After 14 days of incubation at 37 °C with 5% CO_2_, Caco-2 cells were washed with PBS three times, incubated with Dil for 15 min with red fluorescence labeling, then washed with PBS for another three times and stained with Hoechst 33342 for 10 min to mark the nuclei. The stained cell samples were divided into two groups, one without any treatment but with a serum-free culture medium and the other co-incubated with CS-TPP-ALG nano-preparation. The two groups of cells were subjected to FRAP assays to examine the difference in cell membrane fluidity. Two images were taken before photobleaching, using 100% transmission at a laser wavelength of 480 nm to bleach a circular area on the plasma membrane. The corresponding recovery of fluorescence intensity in the bleached region was recorded and a bleaching recovery curve was fitted to compare the rate and extent of fluorescence recovery in the cell membrane of the two groups after bleaching.

### 2.12. Establishment of Type 2 Diabetic Rat Model

SD rats were fed high-fat diets containing 45% fat to induce insulin resistance. After one-month diets, a standard 16 h fast in SD rats was conducted, and a 30 mg/mL STZ solution prepared with 0.1 mol/L citrate-sodium citrate buffer (pH 4.5) was injected intraperitoneally at a dose of 30 mg/kg (avoid light, the injection was completed within 30 min). Then the rats were given sufficient water and fasted. Restored the diet after 2 h. If the fasting blood glucose was higher than 16.67 mmol/L for two consecutive times, and the rats were accompanied by significant polydipsia, polyphagia, polyuria, and weight loss, it is considered to be successfully modeled and subsequent experiments could be carried out.

### 2.13. Pharmacokinetics Study of Exenatide Nano-Preparation

For the pharmacokinetics study of CS-TPP-ALG, twelve normal rats were randomly divided into two groups and fasted overnight. Exenatide saline solution (65 μg/kg) was given subcutaneously in group one as a positive control group. CS-TPP-ALG nanoparticles (650 μg/kg) were administrated by gavage in group two. Then, the blood was collected from the orbit at 0.5, 1, 2, 3, 4, 6, 8, 12, and 24 h, respectively, centrifuged at 3000 rpm for 10 min to separate the serum. According to the immunoassay, the concentration of exenatide was determined and the blood-drug concentration-time curve was plotted.

### 2.14. In Vivo Pharmacodynamics Study

To evaluate therapeutic effects, thirty T2DM rats were randomly divided into five groups, fasted overnight, and labeled respectively. Insulin (65 μg/kg) and exenatide (150 µg/kg) were administrated by subcutaneous injection, while CS-TPP and CS-TPP-ALG were administrated by gavage at a dose of 650 µg/kg. Blood samples were collected from the tail tip at 0, 0.5, 1, 2, 3, 4, 6, and 10 h after administration to observe the changes in blood glucose levels, and the blood glucose-time curve was plotted.

## 3. Results and Discussion

### 3.1. Characterization and Morphology Analysis of CS-TPP-ALG

CS-TPP-ALG and TMC-TPP-ALG nano-preparations were successfully prepared. The particle size of CS-TPP-ALG was 445.3 ± 2.159 nm and the electric potential was 6.27 ± 0.456 mV, with a PDI of less than 0.15 (Figure 1b). The particle size of TMC-TPP-ALG was 550 ± 41.04 nm and the electric potential was 2.05 ± 0.968 mV, with a PDI of less than 0.15 (Figure S2a). The morphology of CS-TPP-ALG (Figure 1c) and TMC-TPP-ALG (Figure S2b) was quasi-spherical, with a particle size of approximately 500 nm and a relatively uniform distribution. Additionally, the particle size and TEM images of CS-TPP and Rho-NPs were supplemented in Figure S3.

### 3.2. In Vitro Release Study

To verify the protective effect of formulation on the encapsulated exenatide under low pH conditions of gastric acid, the release of CS-TPP-ALG in acidic simulated gastric fluid and alkaline simulated intestinal fluid was investigated. As shown in Figure 1d, the release of exenatide from CS-TPP-ALG in SIF reached 90% in 1 h, while that in SGF was less than 10% within 3 h due to the gelation properties of sodium alginate in an acidic environment [29], suggesting that sodium alginate enteric coating can protect chitosan nanoparticles against gastric acid damage, thus functioning effectively for the controlled release.

### 3.3. Determination of Encapsulation Rate and Loading Capacity

The encapsulation rate and drug loading capacity of exenatide chitosan nano-preparation are shown in Figure 1e, the encapsulation rate is (52.92 ± 1.19)%, and the loading capacity is (3.08 ± 0.085)%, indicating that the formulation presents good drug loading performance and subsequent experiments could be carried out.

### 3.4. Cytotoxicity Study

**Figure 2:**
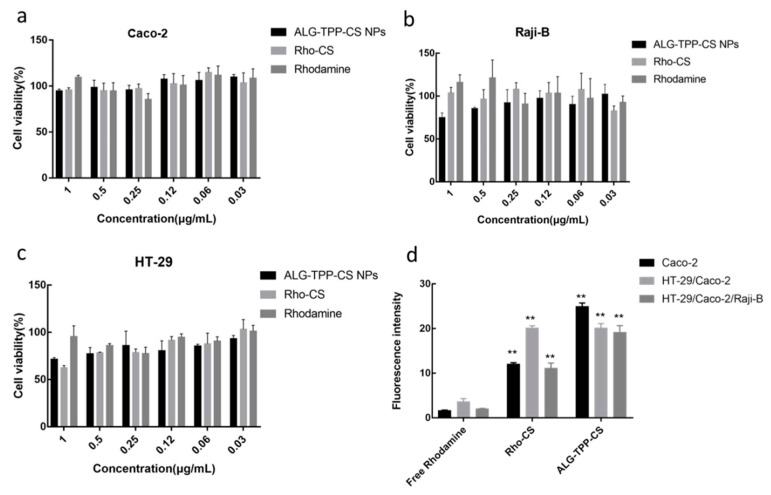
(**a**–**c**) The cytotoxicity of Rhodamine B, Rho-CS and Rho-CS-TPP-ALG NPs in Caco-2 (**a**), HT-29 (**b**) and Raji-B (**c**) cells. (**d**) Cellular uptake of Rhodamine B, Rho-CS and Rho-CS-TPP-ALG NPs in Caco-2, Caco-2/HT-29, Caco-2/HT-29/Raji-B cells. ** *p* < 0.01 (*n* = 3), VS Rhodamine B.

The cytotoxicity of free Rhodamine B, Rho-CS, and Rho-CS-TPP-ALG NPs to Caco-2, HT-29, and Raji B cells was shown in Figure 2a–c. The results showed that free Rhodamine B, Rho-CS, and Rho-CS-TPP-ALG NPs had no cytotoxicity to Caco-2, HT-29, and Raji B cells, indicating that the nano-formulation and Rhodamine B have good biocompatibility and biosafety at the cellular level.

**Figure 3:**
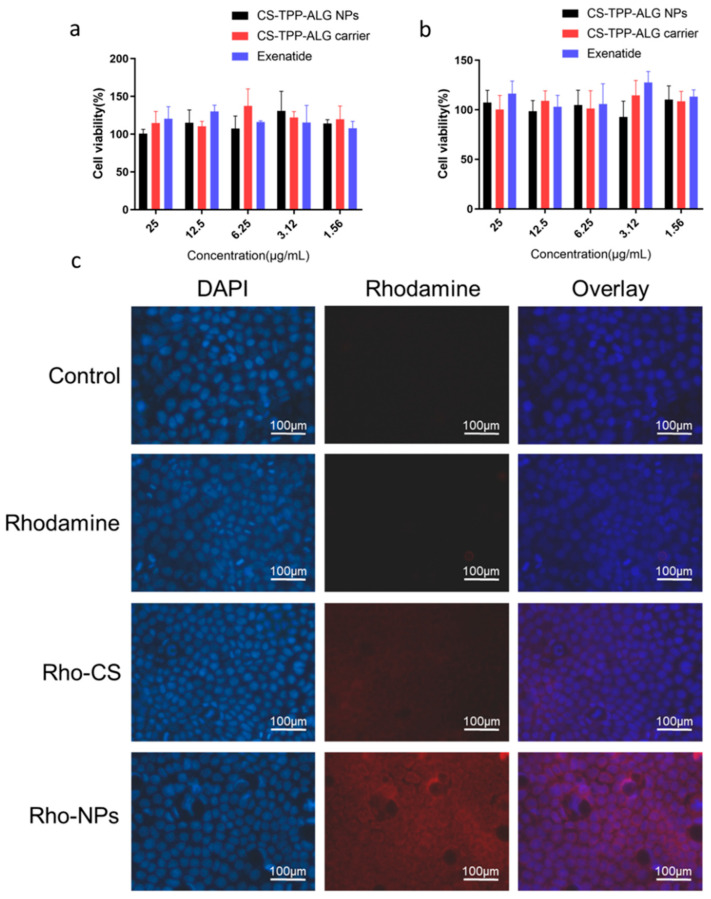
(**a**,**b**) The cytotoxicity of CS-TPP-ALG NPs, CS-TPP-ALG carrier, and exenatide in Caco-2 cells after 24 h (**a**) and 72 h (**b**). (**c**) Intracellular distribution of Rho-CS-TPP-ALG NPs in Caco-2 cells. Cells were exposed to Rhodamine B, Rho-CS, and Rho-CS-TPP-ALG NPs at 37 °C for 4 h, the concentration of fluorescence was 1 µg/mL.

As shown in Figure 3a,b, the cell viability of CS-TPP-ALG drug-loaded nanoparticles, CS-TPP-ALG blank carrier, and exenatide on Caco-2 at 24 h and 72 h were close to 100%, and the cells remained in a growth stage, indicating that CS-TPP-ALG drug-loaded nanoparticles, CS-TPP-ALG blank carrier, and exenatide were of high safety, and could be used in the subsequent experiments.

### 3.5. In Vitro Cellular Uptake of Exenatide Nano-Preparation

The intracellular uptake of different exenatide nano-preparations was measured by flow cytometry (Figure 2d). When the fluorescence concentration was 1 µg/mL, the intracellular uptake of Rho-CS-TPP-ALG NPs was significantly higher than that of free Rhodamine B and Rho-CS groups, indicating that the preparation had the best cellular uptake ability. Meanwhile, the intracellular uptake of Rho-CS was also significantly higher than that of free Rhodamine B. With the positive charge of chitosan, it is conducive to the absorption and crossing of the negatively charged membrane, facilitating the entry of agents into the cell [30,31]. Moreover, the intracellular uptake of Rho-CS-TPP-ALG NPs was further elevated in comparison with the Rho-CS group, which was imparted by the enteric coating of sodium alginate. Attributed to its favorable adhesiveness, sodium alginate can prolong the residence time on the mucus layer and promote the transcellular absorption of drugs [32,33].

### 3.6. Intracellular Distribution of Exenatide Nano-Preparation

**Figure 4:**
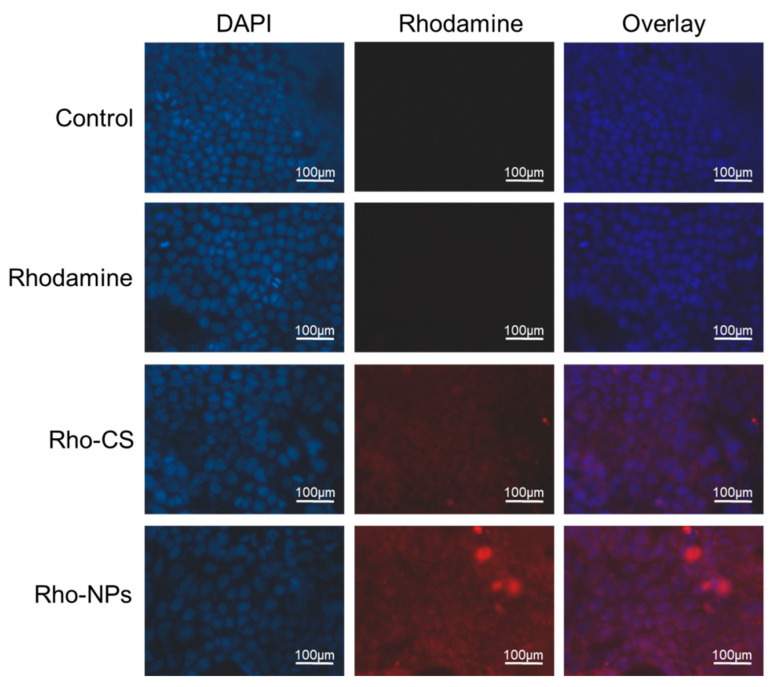
Intracellular distribution Rho-CS-TPP-ALG NPs in Caco-2/HT-29 cells. Cells were exposed to Rhodamine B, Rho-CS, and Rho-CS-TPP-ALG NPs at 37 °C for 4 h, the concentration of fluorescence was 1 µg/mL.

The distributions of Rho-CS-TPP-ALG NPs in cells co-incubated with Caco-2 (Figure 3c) and Caco-2/HT-29 (Figure 4) for 4 h were examined using the fluorescence microscope. The results indicated that there was neither red fluorescence in the control group, nor obvious red fluorescence in the Rhodamine B group. A small amount of red fluorescence was observed in the Rho-CS group, and the Rho-CS-TPP-ALG NPs red fluorescence was more obvious and concentrated in the cytoplasm.

### 3.7. Effect of Exenatide Nano-Preparation on Fluidity of Caco-2 Cell Membrane

**Figure 5:**
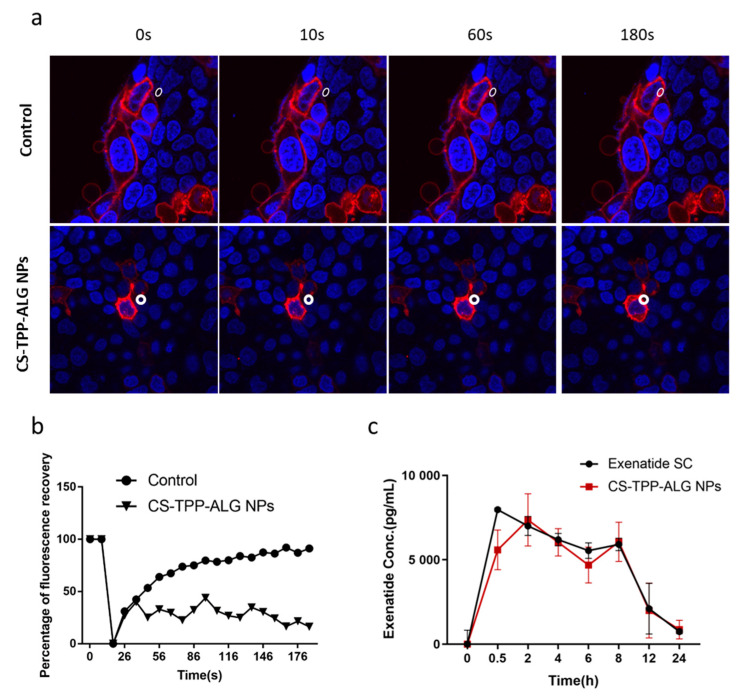
(**a**) Process chart of fluorescence bleaching recovery. White circles indicate the bleaching area on the selected cell membrane. (**b**) Fluorescence bleaching recovery curve. (**c**) Pharmacokinetic curve of exenatide in rats with SC injection of exenatide solution at a dose of 65 μg/kg and oral administration of CS-TPP-ALG NPs at a dose of 650 μg/kg.

The fluorescence bleaching recovery technique was used in this experiment to investigate the influence of exenatide nano-preparation on the fluidity of the Caco-2 cell membrane. Figure 5a,b showed the experimental procedure of fluorescence bleaching recovery, where fluorescence photographs were obtained at different time points before and after fluorescence bleaching in selected areas of the Caco-2 cell membrane. The results indicated that the nano-preparation group showed a tendency to slow down the cell membrane flow, demonstrating that exenatide nano-preparations could promote the absorption of drugs by enhancing the adhesion to cell membranes and slowing down the mobile phase of cell membranes [34].

### 3.8. In Vivo Blood Exenatide Level and Bioavailability

As shown in Figure 5c, exenatide injected subcutaneously reached peak drug concentration at 0.5 h and showed a decreasing trend at the subsequent time, with an area of 35,119 ± 1303 under the curve. Oral administration of CS-TPP-ALG loaded nano-preparation reached peak drug concentration at 2 h, with an area of 32,173 ± 2201 under the curve. The oral bioavailability was calculated to be about 9.16%.

### 3.9. Evaluation of Pharmacodynamic Properties

**Figure 6:**
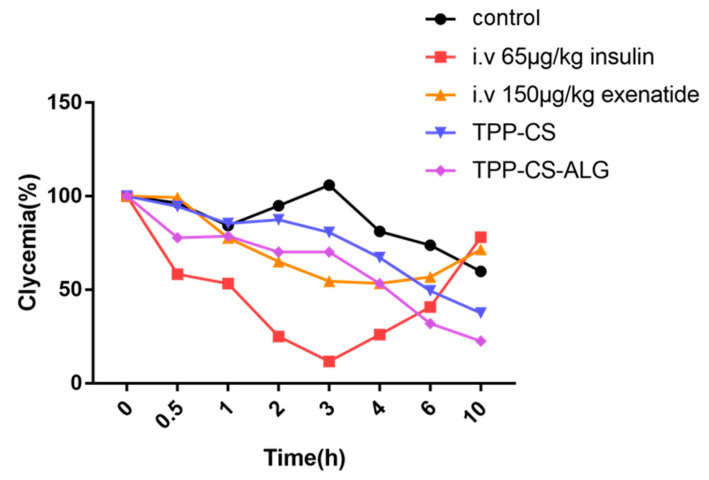
Pharmacodynamics of exenatide in rats with SC injection of exenatide solution at a dose of 150 μg/kg and oral administration of various formulations at a dose of 650 μg/kg.

Figure 6 showed the changes in blood glucose over time in successfully modeled type 2 diabetic rats given different preparations. The oral saline group indicated no hypoglycemic effect, with an increase in blood glucose concentrations at 3 h, followed by a decrease thereafter. In the subcutaneous insulin group (65 μg/kg), the blood glucose concentration decreased significantly within 3 h, reaching a minimum at 3 h, and continued to increase after 3 h. The blood glucose concentration in the exenatide (150 µg/kg) subcutaneous injection group showed a decreasing trend within 3 h, followed by a slow recovery. In the CS-TPP group, the blood glucose concentration decreased gradually after 3 h and still showed a downward trend after 10 h. Compared with the CS-TPP group, the CS-TPP-ALG group exhibited a stronger hypoglycemic effect, with blood glucose continuously declining after 6 h. Therefore, the hypoglycemic effect of the CS-TPP-ALG is long-lasting and has a certain slow-release feature.

In the previous study, TMC-TPP-ALG nanoparticles were successfully prepared. Herein, we proposed further pharmacodynamic studies to evaluate the oral hypoglycemic effect of TMC-TPP-ALG. Figure S2c showed the changes in blood glucose over time in the successfully modeled type 2 diabetic rats after administration of TMC-TPP-ALG nano-preparation. Oral administration of the TMC-TPP-ALG nano-preparation group indicated no significant decrease in blood glucose level, demonstrating that TMC-TPP-ALG had no obvious hypoglycemic effect. A possible explanation is that the quaternized trimethyl chitosan carries a stronger positive charge and is more tightly attached to the drug, which somehow impedes the smooth release of exenatide [20]. On the other hand, the methyl groups attached to TMC cause steric hindrance that partly conceals the positive charge and affects the contact between nanoparticles and cell membrane [35,36]. Consequently, the exenatide concentration in vivo fails to reach the threshold concentration for the oral administration of exenatide to take effect in the rats.

## 4. Conclusions

In this study, the alginate-coated chitosan nanoparticles, with excellent stability and high drug payload, exhibit the great potential to orally deliver the anti-diabetes peptide drug exenatide. To further elucidate the transmembrane mechanism and in vivo efficacy of the preparation, its physicochemical properties and kinetic behaviors were investigated in detail. The coating with alginate improves the anti-acidic property and the mucoadhesion of the nano-preparation, as evidenced by the in vitro release study and the FRAP assay. Transmembrane permeability analysis revealed that CS-TPP-ALG NPs had the best cellular uptake ability, consistent with the evaluation in rats, which indicated the good hypoglycemic effect of the chitosan-based nanoparticles. Considering the favorable effect of the positive charge carried by chitosan in promoting the transport of exenatide, an alternative carrier was constructed using derivative trimethyl chitosan. While the in vivo pharmacodynamics study indicated that the stronger positive charged TMC-TPP-ALG NPs did not show a good hypoglycemic effect, reflecting that in addition to the charge of the carrier, the hydrophilicity and hydrophobicity of the material, the spatial structure, and the ability to interact with the cell membrane may have a collective impact on the gastrointestinal absorption of the peptide. Taken together, natural chitosan-based nanocarrier is a promising strategy and practicable template for the oral delivery of various therapeutic agents, presenting broad application prospects. While current research in vitro and in vivo is still in the validation stage, the utilization of chitosan-based nanoparticles in the clinic still has a long way to go.

## Data Availability

Data is contained within the article or Supplementary Materials.

## Supplementary Materials

The following supporting information can be downloaded at: https://www.mdpi.com/2073-4360/14/11/2181/s1, Figure S1: (a) High performance liquid chromatography conditions. (b) Standard curve of exenatide. (c–d) Specificity: HPLC chromatograms of methanol (c) and exenatide (d), Figure S2: (a) The size distribution and zeta potential of TMC-TPP-ALG. (b) TEM image of TMC-TPP-ALG. (c) Pharmacodynamics of exenatide in rats with oral administration of TMC-TPP-ALG at a dose of 650 μg/kg, Figure S3: Characterization and morphology analysis of Rho-NPs and CS-TPP. (a) The size distribution and zeta potential of Rho-NPs. (b) TEM images of Rho-NPs. The scale bars of the left figure and right figures are 2 μm and 500 nm, respectively. (c) The size distribution and zeta potential of CS-TPP. (d) TEM image of CS-TPP. The scale bar: 200 nm.
