# Correction: Yang et al. Construction and Evaluation of Chitosan-Based Nanoparticles for Oral Administration of Exenatide in Type 2 Diabetic Rats. *Polymers* 2022, *14*, 2181

**DOI:** 10.3390/polym15132852

**Published:** 2023-06-28

**Authors:** Jian-Miao Yang, Lin-Jie Wu, Meng-Ting Lin, Yi-Ying Lu, Tian-Tian Wang, Min Han, Bin Zhang, Dong-Hang Xu

**Affiliations:** 1 Department of Pharmacy, The Second Affiliated Hospital, College of Medicine, Zhejiang University, Hangzhou 310009, China; yangjm@enzemed.com (J.-M.Y.); 15574881635@163.com (T.-T.W.); 2 Taizhou Hospital of Zhejiang Province, Zhejiang University, Taizhou 317099, China; 3 Institute of Pharmaceutics, College of Pharmaceutical Sciences, Zhejiang University, Hangzhou 310058, China; 22119114@zju.edu.cn (L.-J.W.); 15700060560@163.com (M.-T.L.); 21919089@zju.edu.cn (Y.-Y.L.); 4 Zhejiang Strong Pharmaceutical Co., Ltd., Hangzhou 311500, China

The authors wish to make the following corrections to this paper: [1].

There was a mistake in Figure 3 as published. Figure 3c must be substituted with the following one. The corrected Figure 3 is presented below.

The authors state that the scientific conclusions are unaffected. This correction was approved by the Academic Editor. The original publication has also been updated.

## Figures and Tables

**Figure 3:**
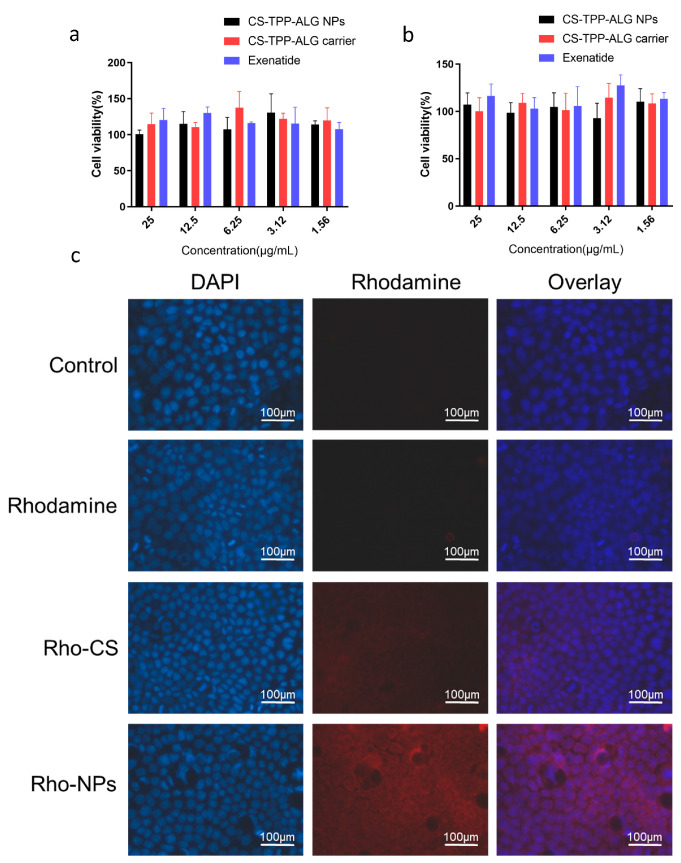
(**a**,**b**) The cytotoxicity of CS-TPP-ALG NPs, CS-TPP-ALG carrier, and exenatide in Caco-2 cells after 24 h (**a**) and 72 h (**b**). (**c**) Intracellular distribution of Rho-CS-TPP-ALG NPs in Caco-2 cells. Cells were exposed to Rhodamine B, Rho-CS, and Rho-CS-TPP-ALG NPs at 37 °C for 4 h, the concentration of fluorescence was 1 µg/mL.
