# Supplementary material for: Construction and Evaluation of Chitosan-Based Nanoparticles for Oral Administration of Exenatide in Type 2 Diabetic Rats
Source: Polymers (Basel). 2022 May 27;14(11):2181. doi: 10.3390/polym14112181 (PMC9183037; doi:10.3390/polym14112181)
Supplement: Supplementary file 1 [file polymers-14-02181-s001.zip › polymers-1706989-supplementary.pdf]

Supplementary Information

Construction and Evaluation of Chitosan-Based Nanoparticles for Oral Administration of Exenatide in Type 2 Diabetic Rats

Jian-Miao Yang [1,2,†], Lin-Jie Wu [3,†], Meng-Ting Lin [3], Yi-Ying Lu [3], Tian-Tian Wang [1,3], Min Han [3,*],
Bin Zhang [4,*] and Dong-Hang Xu [1,*]

Department of Pharmacy, The Second Affiliated Hospital, College of Medicine, Zhejiang University, Hangzhou 310009, China; yangjm@enzemed.com (J.-M.Y.); 15574881635@163.com (T.-T.W.)
[2] Taizhou Hospital of Zhejiang Province, Zhejiang University, Taizhou 317099, China
[3] Institute of Pharmaceutics, College of Pharmaceutical Sciences, Zhejiang University, Hangzhou 310058, China; 22119114@zju.edu.cn (L.-J.W.); 15700060560@163.com (M.-T.L.); 21919089@zju.edu.cn (Y.−Y.L.)
[4] Zhejiang Strong Pharmaceutical Co., Ltd., Hangzhou 311500, China
* Correspondence: hanmin@zju.edu.cn (M.H.); zhangbin1@cnstrong.cn (B.Z.); xudonghang@zju.edu.cn (D.-H.X.); Tel.: +86-571-88208437 (M.H.); +86-571-87784529 (D.-H.X.)
† These authors contributed equally to this work.

a

	a	b
0 min	100%	0%
0.1 min	72%	28%
25 min	47%	53%
25.1 min	0%	100%
30 min	stop	

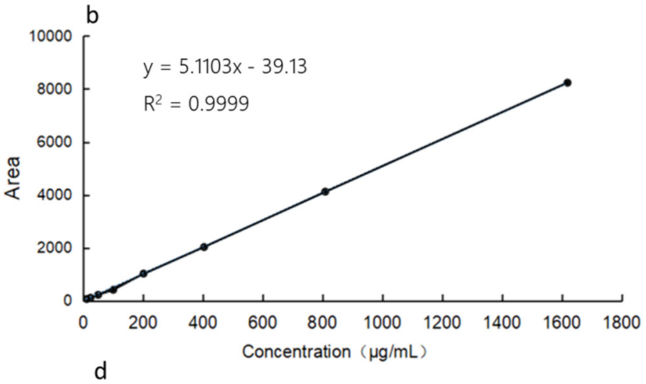

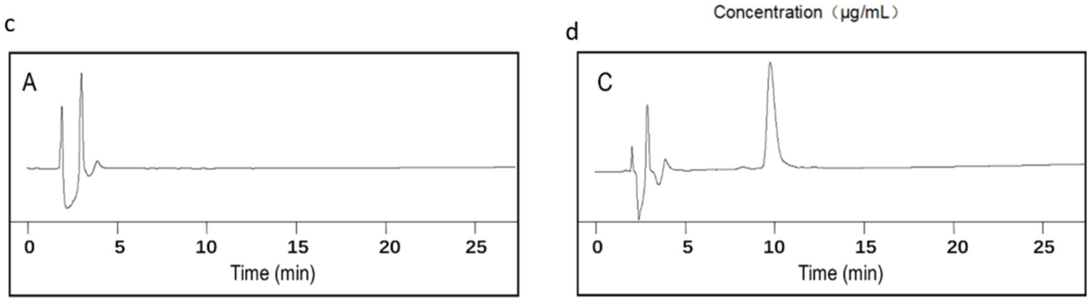

Figure S1. (a) High performance liquid chromatography conditions. (b) Standard curve of exenatide. (c-d) Specificity: HPLC chromatograms of methanol (c) and exenatide (d).

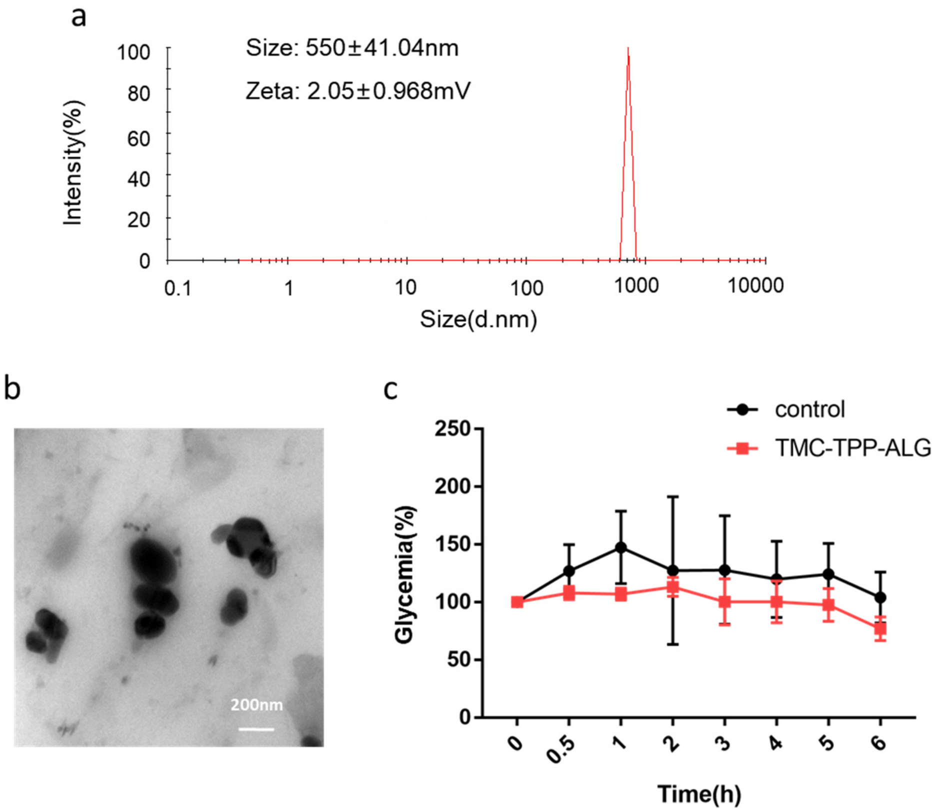

Figure S2. (a) The size distribution and zeta potential of TMC-TPP-ALG. (b) TEM image of TMC-TPP-ALG. (c) Pharmacodynamics of exenatide in rats with oral administration of TMC-TPP-ALG at a dose of 650 μg/kg.

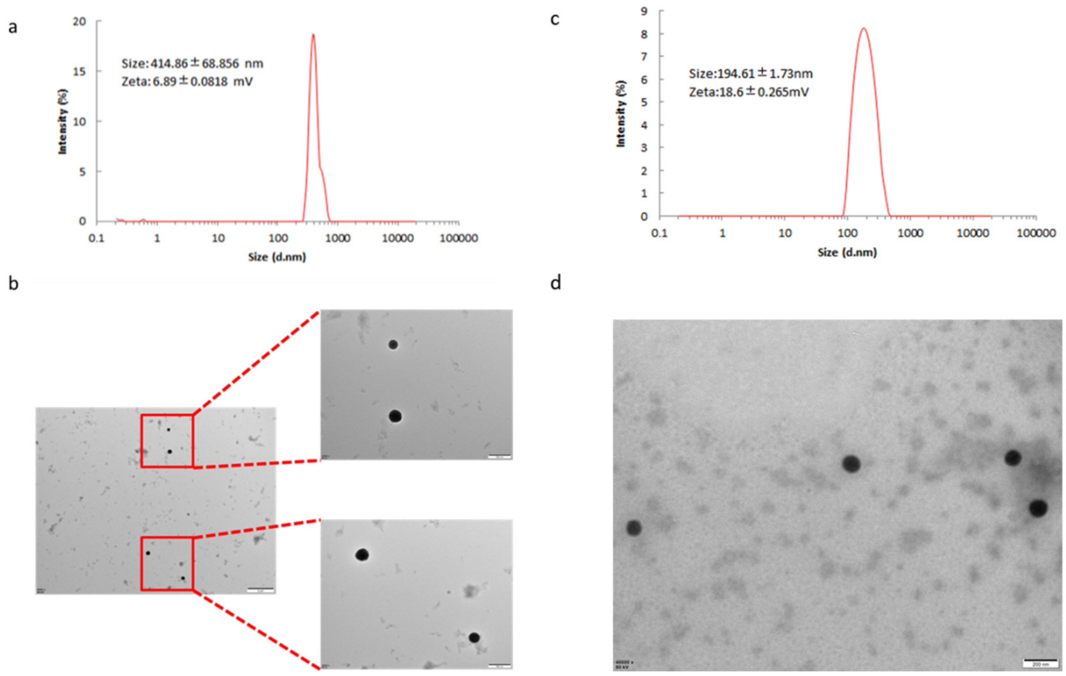

Figure S3. Characterization and morphology analysis of Rho-NPs and CS-TPP. (a) The size distribution and zeta potential of Rho-NPs. (b) TEM images of Rho-NPs. The scale bars of the left figure and right figures are 2 μm and 500 nm, respectively. (c) The size distribution and zeta potential of CS-TPP.(d) TEM image of CS-TPP. The scale bar: 200 nm
